# Household behavior and vulnerability to acute malnutrition in Kenya

**DOI:** 10.1057/s41599-023-01547-8

**Published:** 2023-02-17

**Authors:** Ravi Bhavnani, Nina Schlager, Karsten Donnay, Mirko Reul, Laura Schenker, Maxime Stauffer, Tirtha Patel

**Affiliations:** 1 Geneva Graduate Institute, Geneva, Switzerland; 2 University of Zurich, Zurich, Switzerland; 3 University of Lausanne, Lausanne, Switzerland; 4 Simon Institute for Longterm Governance, Geneva, Switzerland; 5 Columbia University, New York, NY USA

**Keywords:** Politics and international relations, Development studies, Complex networks

## Abstract

Anticipating those most at-risk of being acutely malnourished significantly shapes decisions that pertain to resource allocation and intervention in times of food crises. Yet, the assumption that household behavior in times of crisis is homogeneous—that households share the same capacity to adapt to external shocks—ostensibly prevails. This assumption fails to explain why, in a given geographical context, some households remain more vulnerable to acute malnutrition relative to others, and why a given risk factor may have a differential effect across households? In an effort to explore how variation in household behavior influences vulnerability to malnutrition, we use a unique household dataset that spans 23 Kenyan counties from 2016 to 2020 to seed, calibrate, and validate an evidence-driven computational model. We use the model to conduct a series of counterfactual experiments on the relationship between household adaptive capacity and vulnerability to acute malnutrition. Our findings suggest that households are differently impacted by given risk factors, with the most vulnerable households typically being the least adaptive. These findings further underscore the salience of household adaptive capacity, in particular, that adaption is less effective for economic vis-à-vis climate shocks. By making explicit the link between patterns of household behavior and vulnerability in the short- to medium-term, we underscore the need for famine early warning to better account for variation in household-level behavior.

## Introduction

Acute malnutrition—what effectively puts children at greater risk of mortality, stunts growth, and hinders learning and development (Bhutta et al., 2017; Brown et al., 2020; FAO, IFAD, UNICEF, WFP and WHO, 2022; Headey and Ruel, 2022; Hoddinott et al., 2008; Medialdea et al., 2021; Mullen, 2021; Ramírez-Luzuriaga et al., 2021)—remains a persistent problem in Sub-Saharan Africa. Kenya alone reported 942,000 cases of children aged 6–59 months that were acutely malnourished in the first half of 2022 (IPC, 2022).^1^ As a result of insufficient nutritional intake and absorption, affected children typically lose a significant amount of weight in a short period of time (Brown et al., 2020; WHO, 2013). Changes in the prevalence of acute malnutrition are widely used as an early warning indicator for nutrition-related emergencies and crises (FewsNet, 2020; IPC, 2022).

The ability to identify factors associated with changes in children’s nutritional status is therefore key for interventions aimed at reducing household vulnerability. Yet, when prevalence is gauged to have risen above emergency levels, a humanitarian crisis has most likely materialized (Hillbruner and Moloney, 2012; Maxwell et al., 2020). And while the immediate and basic causes of child malnutrition are well understood, existing famine early warning mechanisms consistently fail to allocate resources where they are most needed, precisely because regional and national nutrition patterns mask meaningful variation at the household level^2^.

We consequently adopt a socio-ecological and coupled natural-human systems lens (Bazilian et al., 2011; Chaffin and Gunderson, 2016; Folke, 2006; Jefferson et al., 2020; Maldonado et al., 2020; Moore et al., 2012; Ostrom, 2009; Schlager and Cox, 2018; van Dijk et al., 2020) to study household food security, defined as “a situation in which all community residents obtain a safe, culturally acceptable, nutritionally adequate diet through a sustainable food system that maximizes self-reliance and social justice” (Hamm and Bellows, 2003, p. 37). Following work in this vein (Balbi et al., 2020; Dobbie et al., 2018; Kaiser et al., 2020) we adopt a perspective that emphasizes the inherent complexity of local food systems—the interaction between household characteristics and behavior, their social, economic and institutional environment, and the dynamic interplay between the two. Formative research has shed light on the key drivers of acute malnutrition risk, yet remains limited in its ability to capture the ‘emergent’ nature of household food security (Becker, 1965; Dercon and Krishnan, 2000; Mohammed et al., 2023; Takeshima et al., 2019; Zingwe et al., 2021)—driven by the interdependencies between ecological and socio-economic sub-systems (Balbi et al., 2020; Ostrom, 2009). We offer two key contributions in this regard. First, we make explicit the salience of household adaptive capacity and heterogeneous coping strategies. Qualitative case studies have analyzed the coping behavior of households in times of food shortages and identified significant differences driven by income (Berg and Emran, 2020; Corbett, 1988; Watts and Bohle, 1993).^3^ More recently, researchers have examined how heterogeneity in latent factors, such as adaptive capacity, interacts with household behavior and food security outcomes in otherwise similar livelihood contexts (Pérez et al., 2016; Sam et al., 2019; Wang and Do, 2023). Whereas this small body of research acknowledges the existence of heterogeneous household behavior, key limitations include the absence of an analytical, forward-looking framework, validation, and generalizability (Mayanja et al., 2022).

Second, researchers typically evaluate how malnutrition rates are affected by specific risk factors, such as climate or economic conditions, yet rarely consider situations in which these factors interact, such as the health and market shocks most recently induced by COVID-19 (see Grace et al., 2022 and Brown et al., 2020 for recent exceptions). Rather than analyzing individual indicators that pertain to climate (Barnes et al., 2020; Lipper et al., 2014; Ndiritu, 2021; Tanner et al., 2015; van der Merwe et al., 2022), conflict (Anderson et al., 2021; Buhaug et al., 2015; Dunn, 2018; Hancock, 2020; Martin-Shields and Stojetz, 2019), the economy (Mekasha et al., 2022; Teachout and Zipfel, 2020; WFP, 2020), intrahousehold decision-making (Mohammed et al., 2023) or the empowerment of women (Aziz et al., 2022; Del Boca and Flinn, 2012; Rao et al., 2019) in isolation, we account for inter-linkages across factors that span multiple levels of analysis.

This paper consequently explores how variation in household adaptive capacity mitigates the combined impact of risk factors, such as extreme weather and COVID-19-induced market shocks, on acute malnutrition in otherwise similar communities.^4^ Why, for instance, are some households more vulnerable to acute malnutrition relative to others? And does a given risk factor have the same effect on all households within a geographical context, or does the nature of risk vary? We address these questions using an agent-based model in which households are the key actors. The model is seeded, calibrated, and validated using a mix of quantitative and qualitative data from West Pokot County in Kenya, but easily generalizes to other contexts. We begin our analysis by establishing the fit between the simulated and observed prevalence of acute malnutrition. We then use the model to make leading-edge predictions of acute malnutrition and to conduct a set of counterfactual, “what-if” experiments. The experiments demonstrate (i) how variation in household adaptive capacity shapes vulnerability to acute malnutrition in the short- to medium-term, and (ii) how the nature of risk, holding exposure constant, varies across households. In a final step, we test the generalizability of the model to other contexts, specifically neighboring Turkana county.

Our findings suggest that there are considerable differences in vulnerability to acute malnutrition across wards in West Pokot over time, necessitating a move to household-level determinants. The scenario-based analyses, which underscore the salience of household adaptive capacity, suggest that increasing the ability of households to adapt to changing conditions can significantly mitigate the impact of climate, and to a lesser extent economic shocks. The potential mitigation effect is larger the more vulnerable the household. Both in- and true-out-of-sample validation point to the robustness of our model, underscoring the value of modeling household-level dynamics for evidence-based decision-making. Taken together, our analyses and findings contribute to famine early warning by making explicit the link between household behavior and vulnerability to acute malnutrition.

The paper is organized as follows. Section “Methods” describes the study area, data sources and processing, and our evidence-driven, computational modeling approach. Section “Results” assesses model fit, based on a set of quantitative performance statistics, and assesses the validity of leading-edge predictions. In the section “Scenario-based forecasts”, a set of scenario-based predictions are used to unpack household-level dynamics in a counterfactual experimental setup. Section “Discussion” discusses the implications of our findings for designing effective interventions at the household level.

## Methods

### Study area

**Fig. 1:**
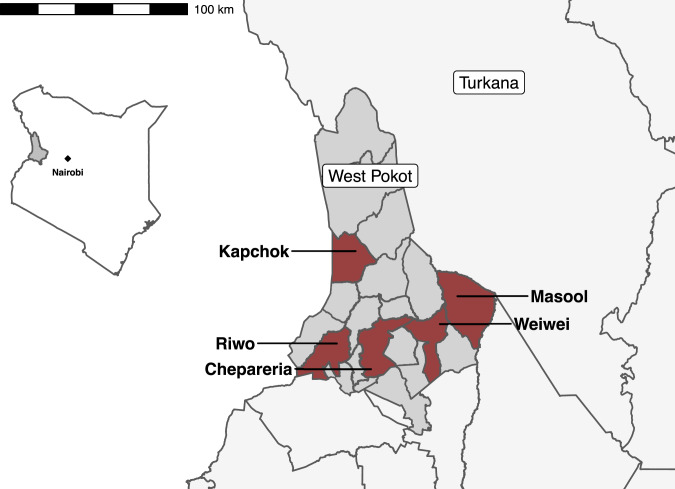
Map of West Pokot County, Kenya. The five wards in our study, colored red, include Chepareria, Kapchok, Masool, Riwo, and Weiwei.

The five study sites located in West Pokot county are depicted in Fig. 1. The region’s three primary producer groups—pastoral communities in the “low lands” (Weiwei and Kapchok wards), agro-pastoralists in higher regions (Riwo and Masool wards), and mixed farmers around the urban center of Chepareria—are equally dependent on the annual long rains. Environmental degradation—a result of prolonged dry seasons followed by long rains and severe floods—is characteristic of the area, with some 90% of maize, the major crop in West Pokot, produced in under rain-fed areas (NDMA, 2023). Poor management of water resources poses yet another challenge to development (Joseph Kanyua, 2020). When droughts diminish food supplies, children typically suffer from malnutrition and stunting. And when water resources decline, children cannot wash their hands, fall sick and become malnourished (UNICEF, 2020). With a majority of people living in similar dwellings and facing similar health and sanitary conditions, it is reasonable to conclude that a high degree of homogeneity exists across household livelihood strategies and adaptive capacities. Yet, we argue to the contrary, suggesting instead that households exhibit variation in their ability to adapt in times of crises—the most vulnerable segments of the population in West Pokot effectively lagging behind given limits on access to critical infrastructure, material, and human resources.

### Data sources and processing

#### Household nutrition surveys

Household nutrition surveillance data were collected by the National Drought Management Authority (NDMA) of Kenya at monthly intervals between January 2016 and March 2020.^5^ The NDMA, in conjunction with local governments, collects data on the nutrition status of children from so-called ‘sentinel sites’—154 localities spread across the 23 arid and semi-arid (ASAL) counties in Kenya. Each site tracks 30 households every month, collecting information on a range of food security measures such as the gender and education of the head of household, income and water sources, local market conditions, and coping strategies. Our dataset features *N* = 14,409 observations of individual children from five sentinel sights in West Pokot county. Supplementary Table S1 online provides summary statistics of the household nutrition surveys. The main anthropometric measure used in this study is the Mid-Upper Arm Circumference (MUAC).^6^ For validation purposes, the data is complemented with three to five interviews per sentinel site (NDMA, 2014). Unlike mass screenings, akin to those conducted for SMART surveys, NDMA surveillance relies on a small number of households at the sentinel site level that is ideally observed repeatedly for a whole year (Maxwell and Hailey, 2020).

#### Stressors

We combine household nutrition survey data with open-source information on climate and food price stressors at equally granular units for West Pokot county (see Hancock, 2020; Martin-Shields and Stojetz, 2019; Mekasha et al., 2022; Ndiritu, 2021; Teachout and Zipfel, 2020; van der Merwe et al., 2022 for the significance of these stressors for predicting acute malnutrition). External stressor intensity is conceptualized as monthly deviations from a long-term average at the ward level. Local market stressors are calculated on the basis of monthly prices for maize provided by the NDMA (2023), using the 10-year average cost of 100 g maize in each local market as a baseline (see Supplementary Fig. S1 online). To model the impact of exogenous climate stressors, we use the information on the seasonal normalized difference vegetation index (NDVI) from the MODIS Vegetation Index (MOD13A3) (Didan, Munoz, Solano and Huete, 2015) (see Supplementary Fig. S2 online). The Supplementary Notes on Data and Model Development online, respectively, detail data construction and measurement, model assumptions and the operationalization of key model parameters.

#### Data matching

We match continuous acute malnutrition rates (in %) based on MUAC observations of individual children and additional household covariates at the ward-year level, with stressors at the county-year level. This yields a total of 21 covariates for acute malnutrition prevalence. All observations are geo-coded at the ward level, drawing on geographical information systems (GIS) and shapefiles from the GADM project (GADM, 2021). For comparability, we translate the continuous acute malnutrition rates for each ward into the 5-point Integrated Food Security Phase Classification Acute Malnutrition scale (IPC III/AMN). The IPC III/AMN classification is an aggregate measure for acute malnutrition rates and is widely used by the humanitarian community to chart escalating degrees of nutrition crisis (IPC, 2021).

These data enable us to generate a model landscape that closely approximates the social and geographic context in West Pokot. Fieldwork was instrumental to “ground-truth” the model and iteratively fine-tune key model mechanisms (see the Supplementary Note on Model Development and Supplementary Fig. S3 online).

### Computational modeling

The computational model presented in this paper was developed under the auspices of the Modelling Early Risk Indicators to Anticipate Malnutrition project (see MERIAM 2018). We utilize an evidence-driven computational modeling approach, a more ‘contextualized’ form of agent-based modeling (ABM) that relies on geographical information systems (GIS) and empirical validation (see Bhavnani et al., 2020 for a discussion of the EDM methodology using examples from the MERIAM project.). The approach combines the methodological advantages of ABM for capturing complex social dynamics and causal relationships while maintaining a high degree of ‘real world’ correspondence to a given study area. As noted by Bhavnani et al. (2020), these kinds of models have been successfully applied to the study of civil violence (Bhavnani et al., 2014; Weidmann and Salehyan, 2013), social inequality (Rogers et al., 2015), neo-patrimonial networks (Geller and Moss, 2008), party competition (Laver and Sergenti, 2011), legislative politics (Laver et al., 2011), and crime (Malleson and Birkin, 2012).

Our model, seeded with data on household characteristics, decisions and behavior, local and more macro-level covariates, analyzes the effect of household-level decisions on acute malnutrition, as well as how households adapt their characteristics and behavior over time, subject to constraints. Multiple rounds of theoretical refinement and empirical validation resulted in the final model design which serves as the best approximation for the sub-national region of West Pokot, Kenya. The following section guides readers through different aspects of the model specification and its development process.

#### Actors

Individual households comprise the primary actors in the model. The model is initially seeded with a set of households to represent the population of the smallest spatial unit for which we have empirical data, i.e., the ward (ADM3). Within each ward, households are spatially distributed to reflect population density and approximate the likelihood of household interaction. The ward level, therefore, constitutes the spatial unit at which we seed the model, generate simulated statistics, and validate model outcomes.

#### Objective and model sequence

**Fig. 2:**
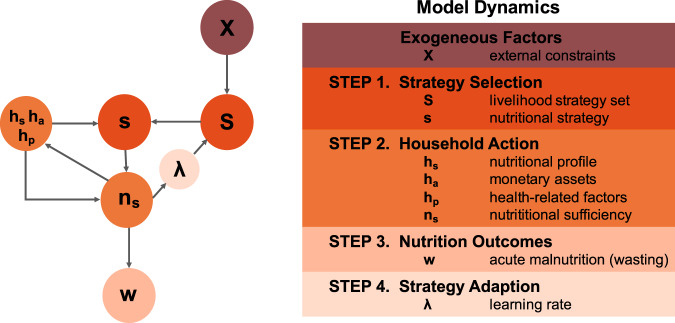
Model specification. The left panel depicts the conceptual flow of the computational model; the right panel provides an overview of model dynamics and variables.

Each household seeks to ensure nutritional sufficiency by selecting and updating nutrition strategies subject to constraints. Specifically, each household repeats a sequence of strategy selection, household action, and adaption in any given model round. This cycle ensures the endogenous evolution of household strategies in response to the behavior of other households, as well as changing external circumstances. Households interact in random sequential order such that each household updates once at a given timestep. Simple linear scaling relates “model time” to real-time intervals and corresponding time-varying model inputs with observed model outputs. Interactions in the model are rule-based, with a stochastic decision process and limited information exchange. The choice of a nutrition strategy is co-dependent, insofar as each household observes and seeks to adopt the ‘best performing’ strategy in their immediate (geographical) context when sufficiency is not attained. Figure 2 provides an overview of the different variables that operationalize the cycle by which individual households update their behavior.

#### Interactions and outcomes

Household nutritional strategies are drawn from a set of available livelihood strategies *S* which determine whether they produce their own food, draw on local networks or buy/barter for goods, or some mix thereof. Strategies are implemented as a vector of three factors—own food production, local networks, buy/barter for goods—where (1, 0, 0), for example, represents a strategy entirely reliant on own food production. Based on our fieldwork, we limit the initial strategy set to plausible combinations of livelihood strategies in a given local context.^7^ As such, the semi-arid lands in Turkana would not enable pastoralist households to engage in agricultural or fishing activities, effectively limiting alternatives for their own food production, a priori reducing their strategy set (see the Supplementary Method online for details of an alternative model specification). Strategies are further constrained by external climatic and economic conditions *X*. Climatic shocks, by way of example, are operationalized as reducing the effectiveness of all strategies that rely on their own food production by a given factor—the smaller this factor, the greater the impact of the shock, e.g. factor 0.2 reduces the effectiveness of the given dimension by 80%.

In each interaction, households randomly select a nutritional strategy *s* among the available livelihood strategy set *S*, specific to each household. As households update their strategies over time. they may utilize identical strategies, given that successful strategies are more likely to be selected or replicated relative to less successful ones. At the extreme, a household may have only one strategy *s* in its set, although learning and adaption ensure the adoption of potentially more successful strategies over time.

The model assumes that the selected nutritional strategy *s* together with the household’s nutritional profile *h*_s_ determines food availability at any given time. *h*_s_ captures whether a given household actually has the ability to produce their own food, draw on local networks or buy/barter for goods, i.e., a strategy is only successful and ensures nutritional sufficiency *n*_s_ if it matches a household’s abilities. And since the availability of food is necessary but not sufficient to ensure (child) nutrition, *n*_s_ is further constrained by health-related factors *h*_p_ and monetary assets *h*_a_. We operationalize both as multiplicative factors ([1… 0]), i.e., the more sick the child or the smaller the assets, the greater the impact on *n*_s_.

We determine a household’s nutritional sufficiency relative to a global nutritional sufficiency threshold *n*_0_, i.e., if the sum of food available per child through own production, local networks and buy/barter for a given household falls under that threshold, it is considered nutritionally insufficient.^8^ Note that calculating nutritional sufficiency per child for a given household ensures that the simulated statistics align with the empirical measurements we draw on. We define acute malnutrition *w* per household as sustained insufficiency, i.e., households fail to meet the nutritional sufficiency threshold *n*_0_ over consecutive rounds. And acute malnutrition prevalence *W* at the ward-month level then is simply the aggregate statistics over all households in a given ward.

#### Strategy adaption

If a household fails to achieve its primary objective of ensuring nutritional sufficiency in a given round of updating, it adapts by selecting a different livelihood strategy *s* in the following iteration, based on the behavior of other households and in response to changing external circumstances. Household adaptive capacity, or learning *λ*, reflects access to developmental endowments, such as critical infrastructure and education, which can prohibit or facilitate the adoption of alternative strategies. Learning is based on a simple “hill-climbing” heuristic where actors tend to adopt those livelihood strategies that are better performing for their local (geographic) neighbors with rate *λ*, independent of their own nutritional profile *h*_s_.

#### Model initialization and calibration

Household nutritional profile *h*_s_ and health-related factors *h*_p_ are specified using monthly nutritional surveys (NDMA, 2023), as are the ward-level malnutrition prevalence rates which are based on MUAC. The number of children in the model directly represents the number of observations covered in the NDMA survey—14,409 observations in West Pokot (see the Supplementary Note on Household Nutrition Surveys online for details that pertain to sample size). We use cross-sectional data on average wealth or food-related household assets to measure monetary assets, *h*_a_, permitting us to generate a distribution of rich vs. poor households in a given area. We combine this household-level data with monthly, ward-level information on extreme weather, specifically the greenness of terrain (NDVI) and changes in market prices of maize to empirically specify the contextual factors *X*.

The simulation is initialized with a random draw among the entire livelihood strategy set *S* available to all actors. Model calibration is then used to estimate household adaptive capacity, in particular, their learning rate *λ*. The model allows for sub-optimal behavior in terms of how households evaluate their food security and choose from a set of available strategies, and demonstrates how these might lead to different nutrition trajectories over time. The geographic embeddedness of the model and use of household survey data allows us to model acute malnutrition prevalence *W* at the ward-month level and compare simulated outcomes to those observed empirically.

A mix of quantitative and qualitative information was used to refine and “ground-truth” the model. In addition, to survey data, we conducted interviews and focus-group discussions with households, community group leaders, and nutritionists in West Pokot and elsewhere to systematically validate model mechanisms and set ranges for parameter specifications (see the Supplementary Method on Qualitative Model Validation online). Model calibration was used to obtain estimates for parameters that are (i) of central relevance to the mechanism in question, and (ii) empirically unobservable.^9^ The household learning rate *λ* ([0.01 0.1]), which dictates the probability that households switch between strategies, the spawn rate ([0.01 0.1]) for new strategy generation, and the mode of learning ({“hill-climbing”}) which determines how households select between strategies together capture the key, yet unobservable aspects of household decision-making.

Seeding model inputs and validating model outcomes with empirical data throughout the simulation ensures close correspondence with real-world dynamics and effectively captures some of the key trade-offs in the behavioral choices that households make to ensure nutritional sufficiency. More specifically, the model captures (i) how strategy selection is constrained by household capacity, (ii) how overall coping strategies are influenced by combinations of external, climatic and economic, constraints, and (iii) how variation in learning or adaption to best-performing strategies shapes outcomes. It is precisely in this regard that we try to capture the inherent complexity of household behavior and decision-making, identifying household strategies *S* or learning rates *λ* that best capture observed empirical dynamics.

## Results

### Model validation

In assessing model performance, both for calibration and validation, we rely on systematic estimations of bias—such as mean-squared difference. In addition, metrics for predictive accuracies—such as the F1-score and Hamming loss function—allow us to assess the degree to which empirical variation in prevalence rate (categories) can be explained by the computational model. We use these measures of in-sample validity as joint indicators of our model’s ability to provide valid predictions (see the Supplementary Method on Quantitative Model Validation online).

In addition, we test the performance of our model on data it was not initially trained on. This includes additional validity tests using standard split-sample approaches but also ‘true’ out-of-sample testing. Specifically, we will rely on leading-edge predictions for 4-month prediction horizons. This approach is designed to estimate short-term future malnutrition outcomes, capturing the likely magnitude of a future crisis and informing stakeholders about appropriate standard short-term responses. In a final step, we test model performance in neighboring Turkana county to assess its generalizability to other contexts.

Table 1 provides a summary of validation exercises and corresponding model performance statistics.

**Table 1:** Summary of validation exercises and quantitative model performance statistics.

Validation Type	Sample	Test Metric	Value	Value Range
Calibration (In-Sample)	West Pokot, 2017–2018	F1	0.72^∗^	0–1
		Hamming Loss	0.25	1–0
		RMSD	0.01^∗^	>0
Temporal Split (Out-of-Sample)	West Pokot, 2017	F1	0.55^∗^	0–1
		Hamming Loss	0.45	1–0
		RMSD	0.01^∗^	>0
	West Pokot, 2018	F1	0.51^∗^	0–1
		Hamming loss	0.49	1–0
		RMSD	0.02^∗^	>0
4-Month Leading-Edge (True Out-of-Sample)	West Pokot, 2019 (Jan.-Apr.)	F1	0.72^∗^	0–1
		Hamming Loss	0.26	1–0
		RMSD	0.02^∗^	>0
	West Pokot, 2019 (May-Aug.)	F1	0.67^∗^	0–1
		Hamming Loss	0.32	1–0
		RMSD	0.04^∗^	>0
	West Pokot, 2019 (Sep.-Dec.)	F1	0.33	0–1
		Hamming Loss	0.56	1–0
		RMSD	0.03^∗^	>0
Generalizability (True Out-of-Sample)	Turkana, 2017–2018	F1	0.52^∗^	0–1
		Hamming Loss	0.48	1–0
		RMSD	0.14	>0

The joint validity criteria include systematic estimations of bias (RMSD) and metrics for predictive accuracy (multiple category F1, Hamming loss score). RMSD measures the degree of deviation of predictions from observed values, with higher values indicating a larger deviation. The cut-off value for this study is 0.2, such that if the RMSD is smaller relative to this value the prediction is valid. Taking into account the imbalanced distribution of observations and the multi-category classification, we find the random prediction baseline in these cases to lie between F1 values of 0.3 and 0.4. We, therefore, set our joint validity criteria such that a model with F1 > 0.5 for internal validity predictive power is successful. Validation samples that fulfill these criteria are marked with an asterisk. Hamming-loss scores are reported as a complement to F1 scores.

#### In-sample validation

##### West Pokot, calibration

**Fig. 3:**
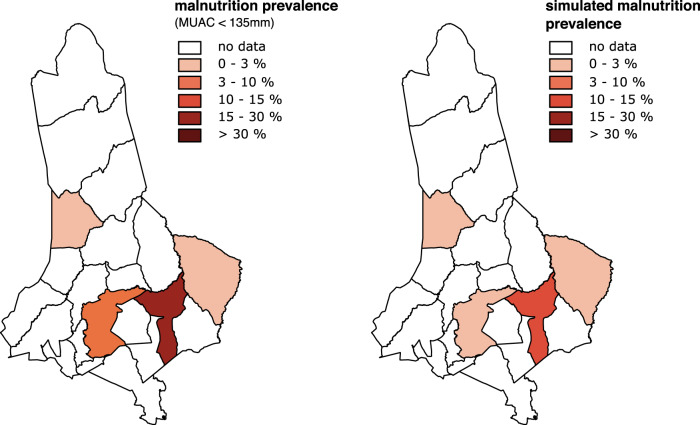
Observed vs. simulated malnutrition prevalence in West Pokot (2017–18). The left panel depicts observed malnutrition prevalence; the right panel depicts simulated malnutrition prevalence. Prevalence is measured using the MUAC measure, and is displayed at the ward-level.

In-sample validation metrics are calculated for a comparison between the distribution of predicted categories vs. outcomes from empirical data. Figure 3 illustrates observed prevalence in the four study areas in West Pokot between 2017 and 2018, using the five-point IPC classification scheme (left picture) and simulated prevalence using the optimized computational model (right picture).

The model produces unbiased predictions of observed acute malnutrition prevalence—also at highly granular spatial units (RMSD = 0.01). To systematically assess bias, we use the average deviation of model predictions from observed malnutrition prevalence, with larger average (root-mean-square) deviations (RMSD) indicating larger bias. In the absence of a standard cut-off, the value for this study is set at 0.2, i.e. if the RMSD is smaller relative to this value, the prediction is valid. In addition to systematic estimations of bias, we analyze predictive accuracy to assess if the model can successfully predict malnutrition prevalence.

We assess model accuracy based on the macro F1-score, i.e. the average of the balanced mean between precision and recall for each category on the five-point IPC III scale. Macro-F1 scores range from 0 to 1, with 1 indicating a perfect classifier. The F1-value of 0.72 indicates that our computational model has a low mis-classification rate, despite the fact that a five-point classification into IPC III categories is more difficult relative to simulations of binary outcomes, for instance, if there is acute malnutrition or not. If the number of observations per category is small, and their distribution is imbalanced, with very few observations in higher categories, the model classifier can get a low mis-classification rate simply by choosing the majority class.^10^ To account for this, we use a second metric for model accuracy, that compares the fraction of categories that are incorrectly predicted to the total number of predicted categories. Hamming loss scores (HLS) range from 0 to 1, with 0 indicating a perfect classifier. As depicted in Fig. 3, we identify one incorrect classification in four sites with a Hamming loss score of 0.25. Just like the F1-score, this indicates that model predictions of acute malnutrition are valid.

#### Out-of-sample validation

##### West Pokot, temporal split

Monthly data collected by NDMA permit us to divide the sample longitudinally to compare model results for the year 2017 to those for 2018.^11^ We compare internal validity based on the split sample to internal validity based on the full, “baseline” sample discussed in the previous section. Observations from 2017 are used to train the computational model. Model performance when optimized for the training data is lower but comparable to that of the full baseline model (RMSD = 0.01, F1 = 0.55, HLS = 0.45). We then test the model against data for the same wards as before, but now for observations in the subsequent year, i.e., 2018, and find that the model’s overall performance is robust, albeit with a slight drop in performance and accuracy (RMSD = 0.02, F1 = 0.51, HLS = 0.49).

The difference in the performance of the baseline model and the out-of-sample models serves as an indicator of the model’s sensitivity to different data inputs. Further, it tells us something about the trade-off between an internally valid model that explains a particular empirical pattern well, and an externally valid model that explains variation beyond the particular case it was optimized for.

#### “True” out-of-sample validation

##### West Pokot, leading edge

Excellent longitudinal coverage by the NDMA in Kenya makes it possible to optimize our model “close” to the prediction period and generate leading-edge predictions of acute malnutrition rates to its true out-of-sample predictive power. To arrive at the leading-edge predictions, we train the model on a subset of the data in the preceding year *t* and then forecast acute malnutrition rates for the *t* + 4 months window. After 4 months we record the forecast error, re-estimate the model, and make a new forecast, and so forth. The result is a set of “bootstrapped” forecast errors that provide a robust assessment of model performance for each time horizon and prediction cycle. To assess the sensitivity of our predictions, we use two alternative time-frames, i.e. 2- and 4-month prediction windows.

**Fig. 4:**
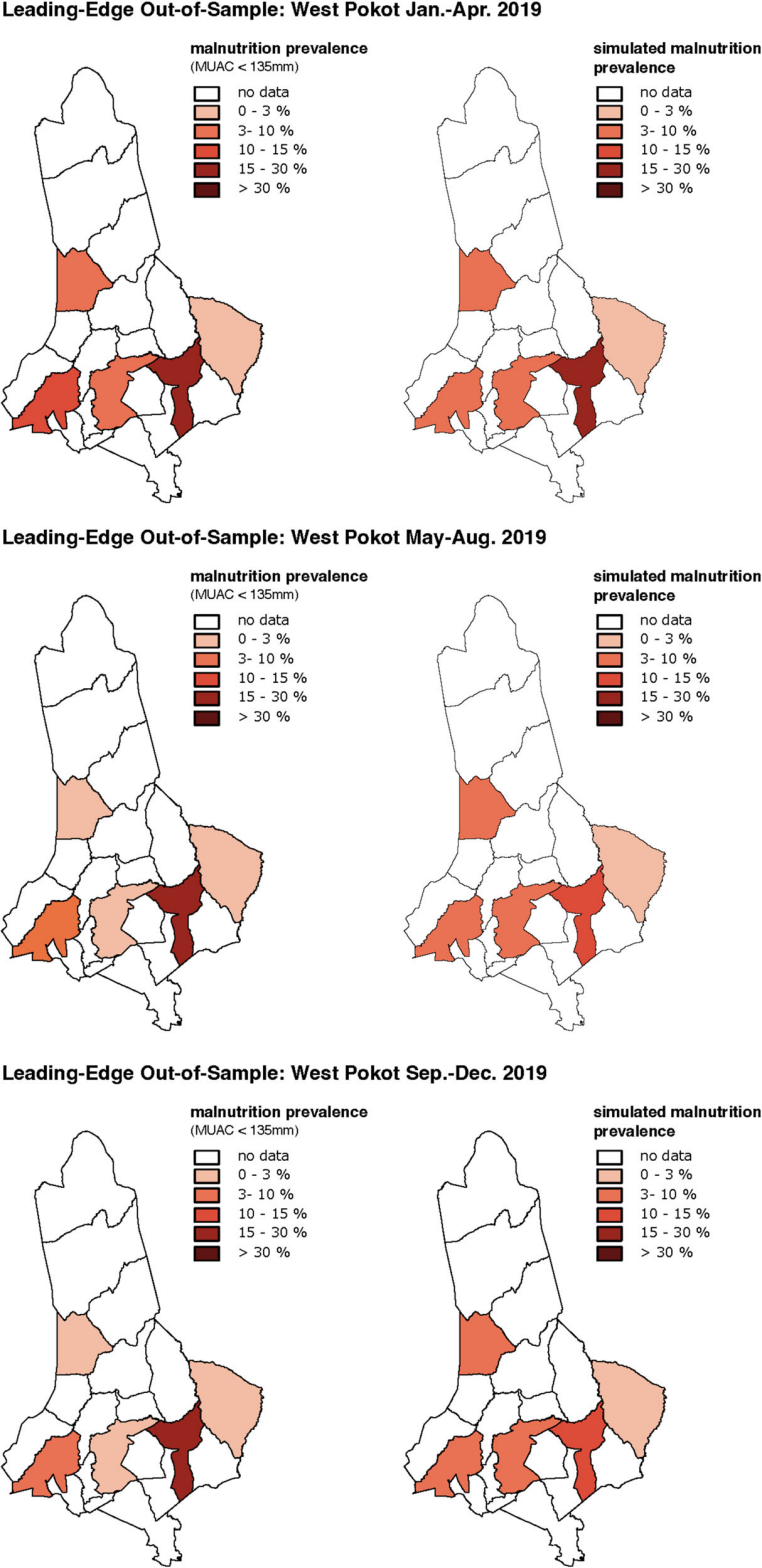
Observed vs. simulated 4-month leading-edge predictions of malnutrition prevalence in West Pokot (2019). Top panel: Jan.–Apr. Middle panel: May–Aug. Bottom panel: Sep.–Dec. Prevalence is measured using the MUAC measure, and is displayed at the ward-level.

Figure 4 illustrates the 4-month leading-edge predictions for West Pokot in 2019, designed to maximize comparability to other forward-looking analyses such as ML1 in Fews Net forecasting cycles. For each of the three prediction periods, observed acute malnutrition prevalence rates (left side) are compared to simulated prevalence rates (right side) (see Supplementary Fig. S4 online for model estimations with a 2-month forecasting window and Supplementary Table S2 online for estimations with both 2-month and 4-month prediction windows).

**Fig. 5:**
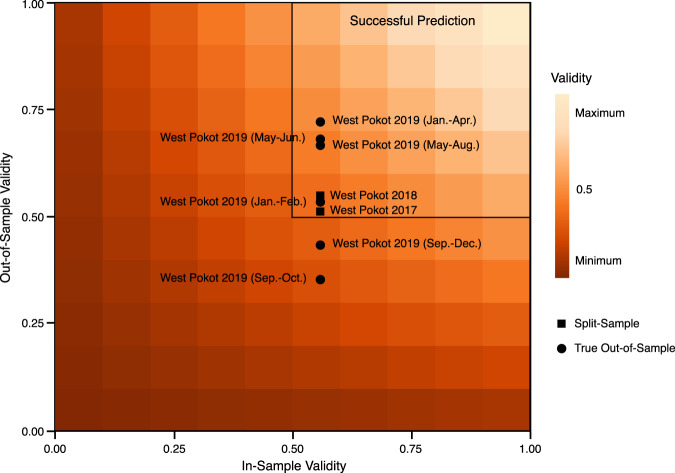
West Pokot: joint validity scale. Simulations with both high in- and out-of-sample validity, measured by F1, are located in the upper-right hand quadrant.

We also evaluate the joint *in-sample* and *out-of-sample* predictive power for each prediction period, as depicted in Fig. 5. The computational model performs best in the first prediction period, January to April 2019 (RMSD = 0.03). After a minor decrease in accuracy for the second prediction window (May–July 2019), the last set of predictions (September–December 2019) is indicative of the weakest model performance.

Here, we observe a significant drop in model accuracy (F1 = 0.43, HLS = 0.56), with an otherwise high degree of quantitative agreement (RMSD = 0.03). Closer inspection reveals that this is an artifact of re-classifying continuous malnutrition prevalence rates per ward (in %) into the 5-point IPC III AMN scale. When aggregating observations, metrics may coincidentally fall into different IPC III categories. Given that we only evaluate five wards, a mismatch in one or two categories drastically lowers the overall predictive accuracy of the model.

##### Turkana, sensitivity analysis

Like West Pokot, neighboring Turkana is among the 23 so-called arid and semi-arid (ASAL) counties in Kenya that are especially prone to extreme weather and climate-induced disasters. The impact of drought, attributable to a delayed onset of the annual long rains in March, and the uneven spatial and temporal distribution of rainfall until August, with a high risk of floods, are felt across both counties. Yet, Turkana is a purely pastoralist region. Instances of cattle raiding and resource-related conflicts are more frequent, relative to the predominantly agro-pastoral West Pokot. Taking into account the differences in livelihood and conflict prevalence by means of alternative parameter specifications (see the Supplementary Method on an Alternative Model Specification online), we use similar household-level data from the NDMA to assess the sensitivity of the model simulations.

**Fig. 6:**
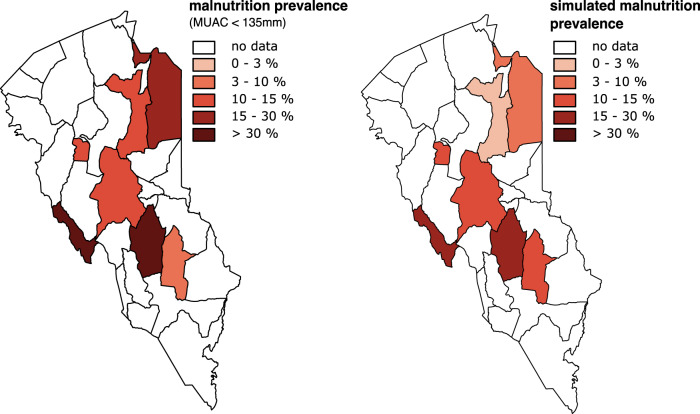
Observed vs. simulated malnutrition prevalence in Turkana (2017–2018). The left panel depicts observed malnutrition prevalence; the right panel depicts simulated malnutrition prevalence. Prevalence is measured using the MUAC measure, and is displayed at the ward-level.

Figure 6 compares observed acute malnutrition prevalence rates (left side) to the simulated values (right side). We find that the simulated values capture a significant portion of the variation in observed acute malnutrition prevalence in Turkana County and generalize well from one to another sub-national context in Kenya, albeit with quantitative model performance significantly lower relative to West Pokot (see the Supplementary Method on Assessing Model Fit in Turkana online).

This occurs for two reasons. First, there is stark, unforeseen variation within Turkana, with some districts facing unforeseen nutritional crises and others much less affected. It is difficult for any model trained on past observations, including ours, to predict acute malnutrition rates that have not been witnessed in the past. Figure 6 highlights, in particular, that the model does not correctly account for the unusually high prevalence rates in the districts of Lokichar and Lokiriama/Lorengippi. And second, performance is lower in Turkana because variation in key indicators such as seasonal conditions cannot fully account for differences across wards, suggesting that other, unobserved dynamics contribute to the greater vulnerability of pastoralist livelihoods in Turkana relative to West Pokot.

### Scenario-based forecasts

Existing early warning mechanisms typically assume that households are endowed with a baseline ability to adapt to changing environmental conditions. However, this may not be the case for the poorest households, as they may have no access to critical infrastructure or education. To assess variation in household adaptation, we compare three scenarios that respectively account for settings where the household ability to learn (*λ*) new strategies is either optimized to fit data on acute malnutrition (baseline), constrained (*λ* decreased by 50%), or enabled (*λ* increased by 50%). We simulate the household’s ability to learn when faced with climate and economic shocks, due to COVID-19 restrictions.

Together with nutrition experts, who helped to validate and refine parameter specifications for each scenario, putting forward the following assumptions (see Supplementary Method on Counterfactual Experiments and Supplementary Table S4 online for an overview of assumptions).

*Climate scenario*: In 2020 a long rain season was expected, which from early summer onward could affect harvest yields and the ability for households to replenish stocks. We model this as equivalent to an early onset of the lean season, which we expect to reduce household availability of food from own production by 20%.

*Economic scenario*: Particularly considering the potential impact of COVID-19, we identify two possible mechanisms: (i) restrictions to mobility and shutdowns which could limit the ability to switch to replacement strategies (especially working in cities); (ii) restrictions to export/import of food (meat in particular). In this scenario, we assume that the ability of households to generate income is reduced by 50%.

**Fig. 7:**
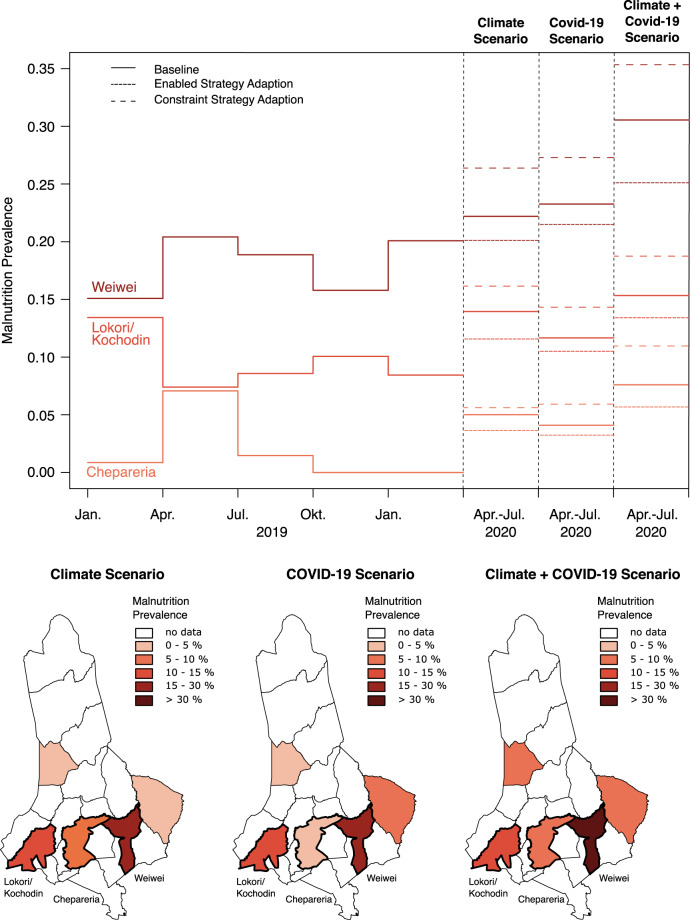
Counterfactual scenarios for select wards in West Pokot (2020). The top panel depicts simulated malnutrition prevalence for climate, Covid-19, climate & Covid-19 shocks from Apr.-Jul. by ward. The bottom panel maps simulated malnutrition prevalence as a result of shocks for the same set of wards. In both panels, prevalence is measured using the MUAC measure.

Given NDMA data coverage to March 2020, the scenario-based forecasts with a 4-month prediction horizon cover the period between April and July 2020. Figure 7 illustrates the simulated acute malnutrition prevalence rates using the fully validated computational model when faced with different combinations of external stressors. The results suggest that already vulnerable wards (and households) in West Pokot tend to become more vulnerable to additional shocks, with the most severe impact in a combined climate and economic scenario. Our findings also suggest that increasing the ability of households to adapt to changing conditions can significantly mitigate the impact, whereas constraining this ability considerably amplifies the impact of the shock. The potential mitigation effect is larger the more vulnerable the household. Lastly, strategy adaption is less effective for economic vis-à-vis climate shocks, given constraints on the number of alternative strategies a household can pursue. Supplementary Fig. S8 online presents the same projections for two other wards covered by the NDMA data, further underpinning the robustness of the model.

## Discussion

Our analysis affords a conceptually richer account of acute malnutrition, relative to existing early warning mechanisms. Correlational models continue to provide predictions at the level of more aggregate spatial and temporal units, effectively masking variability at more granular levels, i.e., across households within the same communities. Adding to the problem, measures used to assess the severity of a crisis—such as the widely-used five-phase IPC food security/acute malnutrition scale—tend to be highly aggregated and largely unsuitable for generating household-level estimates, all the more so given their small sample sizes and correspondingly high levels of sampling variability (Guha and Chandra, 2021). Thus, while composite measures of acute malnutrition prevalence typically assign the same weights and variables to locations within a region (Browne et al., 2014; d’Errico and Pietrelli, 2017; Grace et al., 2022), we underscore the pivotal role of that variation in household behavior plays, modeling differences in the capacity of households to adapt.

Considering the quantitative model performance statistics and the *joint criteria for success*, we are confident that the model captures key dynamics of interest in West Pokot, and generalizes well to the Turkana case. That said, we find considerable differences in household behavior and vulnerability to acute malnutrition across wards in the two study sites. The set of livelihood strategies households use for coping appears to be more narrow in Turkana relative to West Pokot. Purely pastoralist livelihoods and an ongoing nutrition crisis constrain the overall diversity of household strategies—i.e., the mix of available strategies across all households. Low diversity in coping strategies suggests that learning (or adaption) rates were lower in Turkana relative to West Pokot—the broader implication being that it is not necessary for each household to diversify its nutritional strategy, but rather have access to a more diverse set of strategies. Our scenario-based analyses further underscore the salience of household adaptive capacity. Here, we demonstrate that increasing the ability of households to adapt to changing conditions can significantly mitigate the impact of climate, and to a lesser extent economic shocks. We find that external shocks of low magnitude, for instance, small shifts in precipitation patterns, disproportionally affect regions with low diversity of household strategies relative to those with high diversity. In other words, there exist potentially strong feedback loops or cascading effects between the overall diversity of household strategies and seasonal shocks that, all else equal, begin to explain drastic year-to-year changes in malnutrition prevalence across households. These findings closely align with the qualitative insights derived from fieldwork conducted in both regions, effectively highlighting the importance of household strategies and adaptive capacity for mitigating risk.

Our effort builds upon existing approaches to understand and forecast acute malnutrition. The combination of computational modeling, fine-grained nutrition and open-source data enables scalable, evidence-driven prediction at low cost. The approach is also in line with recent calls to view household behavior as part of a complex adaptive system—characterized by co-evolution, the integration of system components at different units of analysis, feedback loops, and nonlinear scaling processes (Bhavnani et al., 2020; Egli et al., 2019; Fraccascia et al., 2018; Naghshbandi et al., 2020; Nasrazadani and Mahsuli, 2020; Siegenfeld and Bar-Yam, 2020).

Yet, our work constitutes a first step in this direction, as it raises difficult questions about the nature and consequences of household behavior and adaptation in times of food crisis. Under what circumstances, for instance, are households more likely to adapt their behavior? Is adaptation more likely to be an independent or collective endeavor, and with what tradeoffs? And what specific measures can be implemented to enhance learning and behavior change? Given that households exhibit variation in adaptive capacity—the latter a prerequisite for community resilience to acute malnutrition—it is imperative to design interventions that more effectively foster opportunities for behavioral changes at the household level.

As the international community increases the spending on emergency food aid each year (Kinyoki et al., 2020), with some 50% of global funds in 2020, approximately $90 billion distributed to nine Eastern African countries, food shortages recur with increased frequency and child acute malnutrition remains widespread (Global Nutrition Report, 2020; Young and Marshak, 2018). Given the endemic nature of the problem, our premise in this paper is altogether straightforward. We argue for more effective targeting of households at-risk and timelier nutrition interventions, with a focus on prevention. Such targeted, evidence-driven intervention may only be achieved if household-level characteristics and heterogeneity in behavior and coping strategies are adequately captured (Guha and Chandra, 2021; Wang et al., 2021).

## Supplementary information

Supplementary Information

## Data Availability

All datasets used in the current study are available via the Harvard Dataverse, a FAIR-compliant data repository: 10.7910/DVN/3NKIKL. Restrictions only apply to the availability of the NDMA household-level data. To safeguard privacy, we provide these data at the ward level, matching the spatial level of aggregation used in this study.

## Footnotes

1 Acute malnutrition is a specific form of undernutrition that is commonly defined as “the combination of wasting and edematous malnutrition, i.e. swelling of the feet and sometimes the lower limbs” (FewsNet, 2006, p. 6).

2 Research on acute malnutrition broadly classifies risk into three categories: (i) immediate causes such as dietary intake and disease at an individual level; (ii) basic causes relating to political and economic conditions at sub-national, country, or international levels; and (iii) under- lying causes such as food security, hygiene environments, care practices, coping strategies and unobservables like adaptive capacity (Mayanja et al., 2022; WHO, 2021; Young, 2020).

3 As such, “the poorest households may not have the same options open to them as those which are better off; for example, they may find it more difficult to obtain credit, may have fewer assets to liquidate, or be more constrained by high dependency ratios in labor migration” (Corbett, 1988, p. 1102).

4 Adaptive capacity is considered to be a “defining feature of complex systems” (Folke, 2006; Nelson et al., 2020).

5 Note that our analysis is constrained by nutrition data availability due to COVID-19—in light of travel restrictions and government guidance on social distancing, NDMA suspended their data collection at the end of March 2020. Since July 2020 NDMA has been using the so-called Family MUAC approach, shifting field monitors’ responsibilities insofar as their main role with this approach is to coach and supervise caregivers, rather than taking the measurements themselves (SOAM, 2020).

6 In times of crisis, the MUAC is often used as the sole anthropometric admission criterion for nutrition programs, taking advantage of its simplicity of use (Collins et al., 2006; Fabiansen et al., 2020). In line with the standard cut-off points suggested by the WHO (2013), we measure acute malnutrition using the most conservative threshold value, *risk* of acute malnutrition (MUAC < 135 mm).

7 We also allow new, similar strategies to ‘spawn’ endogenously with a small rate throughout the simulation to allow for other, potentially more optimal strategies to emerge.

8 The nutritional sufficiency threshold *n*_s_ thus serves to calibrate the overall scale of malnutrition prevalence to the observed empirical levels.

9 We derive results from a parameter sweep with *∼*60,000 parameter combinations optimized for fit with observed prevalence rates as reported by NDMA. Note that the comparatively small sample of four wards is a direct consequence of NDMA’s sampling strategy. Seasonal variation approximated from NDVI data (*X*); no further economic or climate shocks; simulation duration of 365 days simulated in 1000 steps; local household learning (“hill climbing”) with spawn rate 0.03 and learning rate 0.095; household characteristics and health-, food-intake factors (*h*_v_, *h*_p_) seeded from survey data; categories used for household assets *h*_a_ reflect diversity of monetary assets in poor vs. rich households.

10 Correct classification of higher observed malnutrition prevalence, especially Phase 5, is difficult based on the model. This tendency is already visible in Fig. 3, in the pastoral region of Sigor (simulated Phase 2, observed Phase 4).

11 Note that we consider a full year, i.e., a full seasonal cycle. Given that we analyze a representative sample of wards over time, factors such as the overall dispersion of MUAC or the household behavior that produces it are closely comparable. The external distribution of shocks or seasonal patterns may vary between years and thus influence out-of-sample predictive power.
