# Supplementary material for: Household behavior and vulnerability to acute malnutrition in Kenya
Source: Humanit Soc Sci Commun. 2023 Feb 17;10(1):63. doi: 10.1057/s41599-023-01547-8 (PMC9936478; doi:10.1057/s41599-023-01547-8)
Supplement: Supplementary file 1 — Supplementary Information [file 41599_2023_1547_MOESM1_ESM.pdf]

Household behavior and vulnerability to acute malnutrition in Kenya

Supplementary Information

Ravi Bhavnani[1], Nina Schlager[1], Karsten Donnay[2*], Mirko Reul[3], Laura Schenker[1], Maxime Stauffer[4] and Tirtha Patel[5]

[1]Geneva Graduate Institute, Switzerland.
[2]University of Zurich, Switzerland.
[3]University of Lausanne, Switzerland.
[4]Simon Institute for Longterm Governance, Switzerland.
[5]Columbia University, United States.

[*]Corresponding author. Correspondence to: donnay@ipz.uzh.ch

Table of Contents

A Data

A.1 Household nutrition surveys
A.2 Economic stressor data
A.3 Climate stressor data

B Methods

B.1 Computational model development
B.2 Alternative model specification
B.3 Qualitative model validation procedure
B.4 Quantitative model validation procedure

C Results

C.1 Assessing model fit in West Pokot
C.2 Assessing model fit in Turkana
C.3 Counterfactual experiments

List of Supplementary Figures

Figure S1: West Pokot: Market Stressor Intensity (2016–2020).
Figure S2: West Pokot: Climate Stressor Intensity (2000–2019).
Figure S3: Evidence-Driven Modelling Process.
Figure S4: West Pokot: 2-Month Leading-Edge Predictions (2019).
Figure S5: Turkana: 4-Month Leading-Edge Predictions (2019).
Figure S6: Turkana: 2-Month Leading-Edge Predictions (2019).
Figure S7: Turkana: Joint Validity Scale.
Figure S8: West Pokot: Counterfactual Scenarios for Alternative Wards (2019).

List of Supplementary Tables

Table S1: Number of Observed Children per Study Site and Year.
Table S2: West Pokot: Quantitative Model Performance Statistics.
Table S3: Turkana: Quantitative Model Performance Statistics.
Table S4: West Pokot: Assumptions for Counterfactual Experiment.

A Data

A.1 Household nutrition surveys

In Kenya, child anthropometric information is most readily available from SMART Surveys and the annual Kenya Demographic Health Surveys (KDHS), specifically for the years 2003, 2008/9, 2014, and 2021. Whereas the KDHS lacks temporal granularity, SMART surveys are conducted more frequently, albeit with geographical coverage limited to a single county. In a meta–analysis, Brown et al. (2020) identified only five studies in which anthropometric data was taken repeatedly from the same child. In this regard, the household nutrition surveys from the Kenyan National Drought Management Authority (NDMA) are exceptional for their longitudinal coverage. Each month, NDMA samples a small subset of all wards (ADM3), intended to constitute a representative sample for a county (ADM1), and then, in turn, samples households to represent each ward. In combination with open source data on stressors that are also observed in monthly intervals, our study highlights the value of longitudinal nutrition information. Table S1 illustrates the number of observations, i.e. households and MUAC of individual children, per year and study site in West Pokot.

Table S1: Number of Observed Children Per Study Site and Year

Year	2016			2017			2018			2019			2020		
Variable	N	Mean	SD	N	Mean	SD	N	Mean	SD	N	Mean	SD	N	Mean	SD
Study Site	1813			3468			3501			4329			1298		
1	421	23.2%		787	22.7%		801	22.9%		939	21.7%		288	22.2%	
2	447	24.7%		864	24.9%		797	22.8%		893	20.6%		234	18%	
3	326	18%		565	16.3%		681	19.5%		922	21.3%		283	21.8%	
4	369	20.4%		609	17.6%		617	17.6%		681	15.7%		188	14.5%	
5	250	13.8%		643	18.5%		605	17.3%		894	20.7%		305	23.5%	
MUAC	1770	146.107	10.658	3346	146.122	34.62	3321	147.767	30.065	4133	146.376	8.78	1257	148.218	38.873

We calculate child-level statistics rather than household-level statistics. In practice, the two are closely equivalent though as typically only one and not all children in a given household are surveyed at the same time. Nutrition prevalence rates are a fraction of children below the normative wasting threshold in a given sample. While we do not expect missing data for MUAC to skew our results — it only affects 0.6% of observations, spread proportionally across spatial and temporal units (standard deviation of % missing: $\sigma_{ADM3} = 1\%, \sigma_{Years} = 0.3\%$) — missingness on other household covariates goes up to 88% (illness). We impute these values by aggregating household covariates to the ward level, using appropriate rules depending on the scale of measurement. In addition, given the sampling intervals, we calculate any malnutrition statistics only for intervals of three months or longer. This ensures that our measures are certain to cover a full sampling interval and, thus, are not driven by (potentially) biased monthly sub-samples. Finally, we calculate the statistical mean of nutrition prevalence to aggregate from the child- to the ward level. Continuous prevalence rates are reported in the five-point

IPC/AMN scale. We note potential limitations regarding the MUAC measurement as there is significant regional variation. Some argue the MUAC cannot be meaningfully collapsed into IPC categories at all, others do not distinguish between lower IPC categories. In this study, we use similar cut-off points for the MUAC as we would for the WHZ.

A.2 Economic stressor data

To provide an indication of economic stressors in West Pokot, we use data on key food commodities from NDMA's 'County Early Warning Bulletins'. Complementary to nutrition surveys, the NDMA reports the average monthly retail prices of key staple commodities, such as maize, wheat, rice or milk. We analyze changes the cost of maize specifically, as information is most readily available for the largest number of wards over time. We first calculate monthly deviation rates from a three-year average and categorize each month as stressed or not. Second, we define the intensity of market stressors as the mean price increase above the LTA, and z-standardize such that values closer to 0 represent the lowest and values closer to 1 the highest prices in a given year. Figure S1 illustrates the market stressor intensity in West Pokot between 2016 and 2020.

A.3 Climate stressor data

To model the impact of exogenous climate stressors, we use information on the seasonal normalized difference vegetation index (NDVI) from the MODIS Vegetation Index (MOD13A3) (Didan, Munoz, Solano, & Huete, 2015). The NDVI describes 'land fertility' for a region, with the greenest parish being the most food abundant. Therefore, the higher the value of vegetation and soil fertility, the more productive local agricultural and pastoral production. Using this information, we compare (i) each month's statistical value for each area

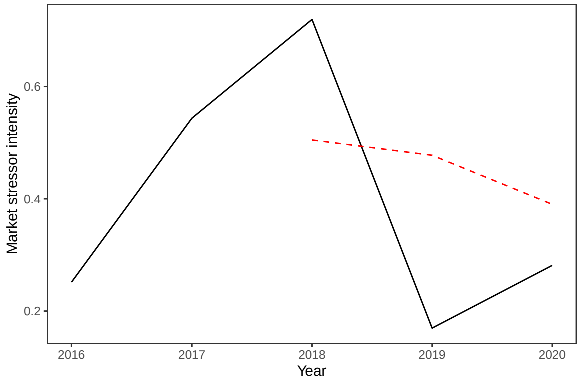

Figure S1: Market Stressor Intensity (2016 – 2020), % Deviation from the 3-Year Rolling Average in Red)

with the average value for all years in that month, and (*ii*) each month's statistical value for each area with the average value for the previous five years in that month to compute anomalies. The approach enables us (*i*) to calculate the intensity of climate stressors and assess their influence on resilience to acute malnutrition in the statistical model, and (*ii*) to explicitly account for seasonal variations in the counterfactual experiment. To assess the sensitivity of our conceptualization of seasonal influences in the counterfactual analysis, we compare the observed NDVI in four wards in West Pokot between 2000 and 2019 to two alternative indicators: temperature and precipitation. As illustrated in Figure S2, we find only small differences among the three indicators and therefore use the NDVI.

B Methods

B.1 Computational model development

Figure S3 illustrates the six-step development process of our evidence-driven computational model as specified in (Bhavnani, Donnay, & Reul, 2020).

(a) Kapchok

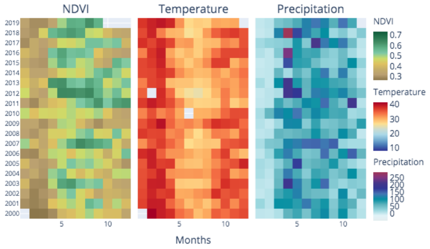

(b) Chepareria

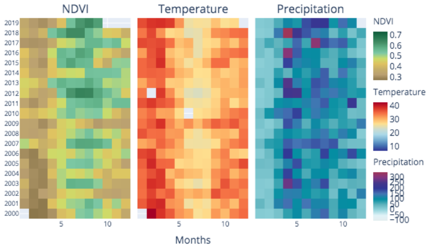

(c) Masool

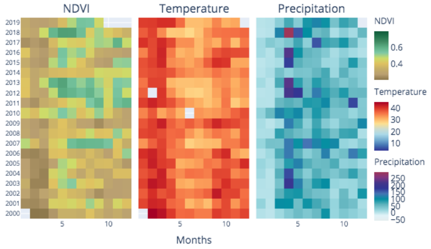

(d) Weiwei

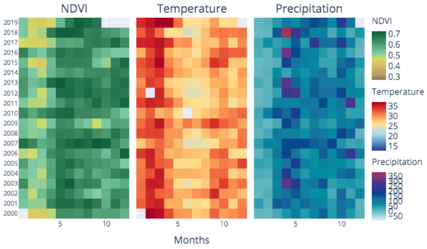

Figure S2: West Pokot: Climate Stressor Intensity (2000–2019)

1. Theoretical Grounding: the model is grounded in existing theoretical and empirical knowledge of the subject matter.
2. Data Construction: relevant data to seed and validate the model are collected, analyzed, and formatted.
3. Model Implementation: a preliminary version of the model is implemented as a computer simulation.
4. Model Refinement and Cross-Case Validation: the model is refined through expert interviews, fieldwork, and out-of-sample testing.
5. Counterfactual Analysis: a valid model is extended by implementing counterfactual, what-if experiments to explore how simulated trends are altered under different conditions.
6. Model Uptake: suggestions to visualize and present model results are put forth in an effort to make the model accessible to relevant end users and stakeholders.

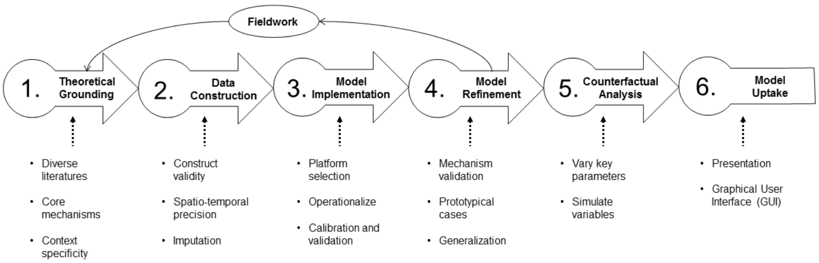

Figure S3: Evidence-Driven Modelling Process (Source: Bhavnani et al., 2020)

Extensive pre-testing, theoretical and empirical validation result in the design of a model "prototype" that serves as a best approximation for West Pokot and may be generalizable to similar cases, further tailored by weighting specific causal drivers, behavioral parameters, and mechanisms. See the next section, B.2, for an alternative model specification, tailored predominantly pastoralist contexts, for instance Turkana county.

B.2 Alternative model specification

To capture the influence of pastoralism as a predominant livelihood strategy in Turkana county, the model is fine-tuned by means of two specific adjustments.

- First, we update the theoretical assumptions for the strategy set (S) in the model, tailoring it better to pastoralist households. We assume that pastoralist households use distinct strategies to cope with acute malnutrition relative to households that rely on agro-pastoralism as their main livelihood. While agro-pastoralist households rely mainly on the diversification of production and income, for example planting other sorts of crops or bee harvesting, pastoralist households are mainly reliant on livestock,

which also increases the likelihood for migration and overall mobility. One could assume that pastoralist households have a *larger* set of coping strategies available. Yet, local experts confirmed that such strategies are increasingly hard to realize for pastoralists due to their reliance of livestock herding. The semi-arid lands in Turkana do not enable pastoralist households to engage in agricultural or fishing activities, effectively reducing the range of coping strategies available to them. We therefore assume that pastoralists have a *smaller* set of strategies to cope with acute malnutrition relative to households with other livelihoods. In the model, the set of available strategies can thus be reduced by a factor ranging from 0 to 1 — where 1 represents a 100% reduction in the household's ability to cope with external shocks.

- Second, we fine-tune the parameter for household assets (h_a) in the model. As that pastoralist households are not sedentary, given their search for fertile herding grounds, they accumulate fewer assets relative to more settled households. We seed this variable with cross-sectional data on average wealth or food-related household assets and simplify it to be binary, such that a household is either poor (in absolute terms) or not. h_a then weights the household food source variable (h_s). In this manner, we avoid further complicating the model dynamics, accounting for heterogeneity in the ability of pastoralist households to acquire food, weighted by assets.

By means of fine-tuning the model as outlined above, we are able to capture the increased vulnerability of pastoralists relative to households with other livelihood strategies, keeping the main mechanisms of the model unchanged.

B.3 Qualitative model validation procedures

As part of our efforts to refine the model, we conducted fieldwork in West Pokot county in July 2019. This 'ground-truthing' exercise was essential to ensure that relevant household behavioral dynamics are accurately reflected in our model. With the help of ACF Kenya, we were able to visit four sub-counties in West Pokot: North Pokot (Kacheliba), South Poktot (Senetwo and Chepareria), West Pokot (Kishaunet and Kapenguria), and Central Pokot (Sigor). ACF Kenya selected and mobilized experts and communities based on priorities that we agreed on in advance. Key selection criteria included heterogeneity in expertise and livelihood strategies, respectively. We administered three types of qualitative data collection instruments during our visit:

- *Focus group discussions.* We conducted three FDGs in West Pokot. One with agro-pastoralists in Chepareria, the second with a mother-to-mother-support group in Senetwo, and the third with a group of pastoralists in Kacheliba. The average group size was approximately around 20 individuals. Due to the high number of participants and their active contributions, we had to adjust our initial set of questions to not go over time and overburden the respondents. Thus, during the 60 minute discussion, we focused mostly on household's understandings of malnutrition causes and processes, the

main influence factors, e.g. seasonality, as well as possible coping strategies. The discussions gave us diverse community viewpoints on household understandings of malnutrition causes and processes, the impact of environmental factors, as well as possible coping strategies.

- *Individual interviews.* Parallel to the FGDs, we conducted an individual interview with an elected chairperson of each group. We asked the community to agree on one person which they wanted to nominate for the individual interview based on their level of experience and status within a community. With these in-depth interviews, we aimed to achieve a deeper understanding of the individual assessments of malnutrition dynamics, as well as the implications of shocks and stressors for specific individuals that are influential within a community and thus likely to be able to influence individual behavior. Our interviewees included the chairwomen of the agro-pastoralists in Chepareria, the mother-to-mother-support group in Senetwo, and the group of pastoralists in Kacheliba. The questionnaire consisted of approximately 50 questions structured according to five categories, ranging from hunger, migration and seasonality, to local infrastructure and WASH. Similarly to the FGD, the administration took approximately 60 minutes.

- *Expert interviews: standardized questionnaires.* We conducted 11 expert interviews and subsequently administered our expert questionnaire in paper form. Experts could be government officials, nutrition experts, regional experts or NGO staff. Our sample *inter alia* includes employees of UNICEF, NDMA and government officials from the Departments of Livestock and Agriculture and Education. During the interview we mostly focused on a modeling exercise to visualize the mental models of the experts, including factors at the household level that could affect nutritional outcomes, the causes and consequences of malnutrition, and how these factors relate to each other. Experts were provided with flashcards to visualize their ideas. The standardized questionnaire could be filled in and handed over to ACF Kenya, who assisted us with the collection.

B.4 Quantitative model validation procedures

Model estimations — in terms of effect size and significance — are based on a trade-off between Type I and Type II errors, which translate into falsely rejecting and falsely failing to reject the null hypothesis that our model does not explain the empirical malnutrition prevalence rates. We discuss this trade-off in the context of *internal validity* — the correspondence between optimized model simulations and empirically observed data — and *external validity* — the correspondence between optimized model simulations and empirically observed data for which the model was not optimized. The quantitative assessment of model fit, both for calibration and validation, includes systematic estimations of bias — such as the root mean-squared deviation. In addition, metrics for predictive accuracy — such as the F1-score and Hamming loss-function — allow us to assess the degree to which empirical variation can be explained by

the model. We use these measures of in-sample validity and predictive power as *joint indicators* of our model's ability to provide valid forecasts.

B.4.1 In-sample validity

We assess the in-sample validity of our model by optimizing the fully-specified and refined model, and comparing simulation results against a set of target statistics from empirical data. We use the root mean-squared deviation (RMSD) to measure the degree of deviation between simulated and empirical MUAC statistics per administrative unit for a given observation period:

$$RMSD = \sqrt{\frac{1}{n}\Sigma\Big(\frac{x_i - y_i}{p_i}\Big)^2}$$

where x is the simulated average MUAC, y the empirically observed MUAC, normalized by p the population density estimate, for administrative unit i. Complementing the measurement of error, we measure the in-sample accuracy of the model. Specifically, we consider the degree to which the model correctly predicts MUAC categories as a measure of goodness of fit (GOF). To this end, we transform empirical and simulated prevalence outcomes into multiple categories and use both a generalized F1-score and normalized Hamming loss-function to calculate the fraction of categories that are correctly and incorrectly predicted respectively. The scores range between 0 and 1, with 1 for the F1-score and 0 for the Hamming loss-function indicating perfect accuracy. A potential disadvantage of the GOF approach is that we relate "success" to abstract categories that may have less meaning on the ground than a MUAC score, whereas an advantage of the approach is that it provides stricter criteria for success than correlation-based measures, since any false positive or false negative prediction is counted as failure. Model optimization then proceeds as follows. Through model enumeration, we identify combinations of model parameters that yield minimal RMSD and maximal GOF of simulated vs. empirical MUAC statistics. The model's parameter space $p = \{p_1, p_2, \ldots, p_i\}$ and the range of value for each parameter are informed both by insights from expert interviews and fieldwork, as well as data availability for each case. As a general rule, model optimization is used to obtain estimates for those parameters that are (i) of central relevance for the mechanism in question, and (ii) are otherwise empirically unobservable. We optimize the following parameters: spawn rate $p_{1v} = [0.01 \ldots 0.1]$ for new strategy generation, the learning rate (λ) $p_{2v} = [0.01 \ldots 0.1]$ which dictates the probability that households switch between strategies, and the mode of adaptation p_{3v}= {"random", "hill-climbing", "reinforcement"} which determines how households select between strategies. These parameters capture the strategic decision-making of households, a key dynamic that is not empirically observable. In addition, a set of global parameters determine how interactions are operationalized in the model: the size of the Moore neighborhood $p_{4v} = [1, 2, 3]$ for a given household, and the duration of a single model simulation in model steps $p_{5v} = [100, 500, 1000]$. We

assess the sensitivity of model outcomes to these choices by means of robustness checks. Second, given a range of possible parameter values for each p, the optimized model is that for which combinations of all possible parameter values p_{1v} p_{2v}...p_{iv} yields the lowest RMSD and highest GOF.

Note that there is no consensus for evidence-driven model simulations on a cut-off value at which the degree of agreement between the optimized model and empirical outcomes is considered substantial or satisfactorily high to consider it internally valid. This is largely due to the relative novelty of the method in social science applications.

B.4.2 Out-of-sample validity

Out-of-sample validity refers to the correspondence between optimized model simulations and empirically observed data for which the model was not optimized. We assess the external validity of the model in two ways. First, we analyze the predictive power of the model by splitting the available data input I into two non-overlapping time periods: one which we use to optimize the model (I_A, "training data" to minimize RMSD and maximize in-sample GOF, "in-sample validation"), and one to assess the fit of the optimized model (I_B, "out-of-sample data"). And second, we assess the extent to which the model is generalizable — in terms of temporal transformability and varying data availability.

Monthly datapoints — as collected by NDMA, lend themselves to a *temporal split*. We divide the sample longitudinally to compare model results for the year 2017 to those for 2018. Note that we consider a full year, i.e., a full seasonal cycle. Given that we analyze a representative sample of wards over time, factors such as the overall dispersion of MUAC or the household behavior that produces it are closely comparable. The external distribution of shocks or seasonal patterns may vary between years and thus influence out-of-sample predictive power. We compare internal validity as based on the split sample to internal validity as based on the full, "baseline" sample discussed below. The difference in performance of the baseline model and the predictive model with empirical input data I and I_B, respectively, serves as an indicator for the model's sensitivity to different data inputs. Further, it tells us something about the trade-off between an internally valid model that explains a particular empirical pattern well, and an externally valid model that explains variation beyond the particular case it was optimized for.

B.4.3 True out-of-sample validity

In addition to in-sample and standard split sample validation, we assess the model's true out-of-sample predictive power. In other words: How well can the model forecast in the future. Forecasts into the future are "true" forecasts that are made for time periods beyond the end of the available data. Model performance as reported on in-sample and split sample validity are likely overly-optimistic. This is because data in the estimation period are used

to help select the model and to estimate its parameters, i.e. the model uses data on both sides of each observation to determine the forecast. A true out-of-sample analysis follows a different logic. We estimate the model based on data for some month t and then construct a forecast of acute malnutrition prevalence rates for months $t + 2$ and $t + 4$. After 2 or 4 months we record the forecast error, re-estimate the model, and make a new forecasts for each time horizon, and so forth. The result is a set of true out of sample forecast errors that provide a robust assessment of model performance. Note that the specification and implementation of such models is demanding, given that they must be re-optimized for each new prediction interval. Forecasting the future in real time — so called "leading edge" predictions, require all the available data for estimation, so that the most recent data is used. In short, it is difficult to properly validate our models if data is in short supply or non-existent. NDMA collects large quantities of nutrition data on a monthly basis. The quality of data — its accuracy, resolution and coverage — is far from satisfying the need for near real-time surveillance required by stakeholders and decision-makers. Yet, Kenya is a comparably data-rich case study, allowing us to explore the predictive power of the model on data it was not optimized for. Using NDMA data for the year 2019, we test three separate leading-edge prediction periods. In each 2019 period — (*i*) Jan. to Apr., (*ii*) May to Aug, and (*iii*) Sep. to Dec.— we test both 2- and 4-month prediction horizons.

B.4.4 Joint validity

The validity of a model is given by the joint ability to simulate empirically observed outcomes *in-sample* and provide *out-of-sample* predictive power. Specifically, a model "successfully" simulates empirical outcomes if both in-sample validity and out-of-sample predictive power place the model in the upper right quadrant, as highlighted in Figure 5 in the main text.

We previously noted that the absence of an established standard to assess the empirical validity of computational models in the social sciences, and have advised against treating any model result as valid at face value based on any single criterion. Also as mentioned,the random prediction baseline for multiple categories is much lower than the standard baseline of 0.5 for binary prediction tasks, given that predicting a larger set of categories correctly, i.e., the five IPC categories, is systematically harder than just classifying, for example, whether a famine condition exists or not. Accounting for the fact that observations are unevenly distributed across different prevalence categories in our empirical setting, we found the random prediction baseline in these cases to lie between GOF values of 0.3 and 0.4 (GOF here as measured by F1-score). We therefore set our joint validity criteria such that a model with GOF > 0.5 for internal validity, and GOF > 0.5 for predictive power are considered successful in this more complex prediction setting.

C Discussion

C.1 Assessing model fit in West Pokot

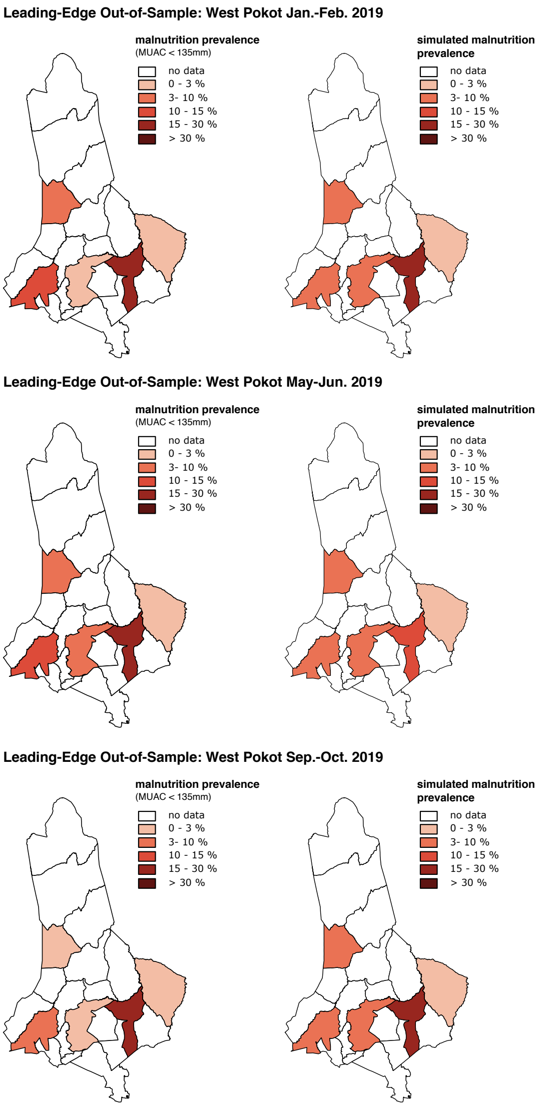

Figure S4: West Pokot: 2-Month Leading-Edge Predictions (2019)

Table S2: West Pokot: Quantitative Model Performance Statistics

Sample	Region	Test Metrics	Results	Range
In-Sample	West Pokot 2017-2018	F1	0.72	$0 \sim 1$
		Hamming Loss	0.26	$1 \sim 0$
		RMSD	0.01	>0
Out-of–Sample (Temporal Split)	West Pokot 2017	F1	0.55	$0 \sim 1$
		Hamming Loss	0.45	$1 \sim 0$
		RMSD	0.02	>0
	West Pokot 2018	F1	0.51	$0 \sim 1$
		Hamming loss	0.49	$1 \sim 0$
		RMSD	0.01	>0
Leading-Edge (2-months)	West Pokot 2019 (Jan.-Feb.)	F1	0.53	$0 \sim 1$
		Hamming Loss	0.45	$1 \sim 0$
		RMSD	0.03	>0
	West Pokot 2019 (May-Jun.)	F1	0.68	$0 \sim 1$
		Hamming Loss	0.31	$1 \sim 0$
		RMSD	0.04	>0
	West Pokot 2019 (Sep.-Oct.)	F1	0.36	$0 \sim 1$
		Hamming Loss	0.62	$1 \sim 0$
		RMSD	0.03	>0
Leading-Edge (4-months)	West Pokot 2019 (Jan.-Apr.)	F1	0.72	$0 \sim 1$
		Hamming Loss	0.26	$1 \sim 0$
		RMSD	0.02	>0
	West Pokot 2019 (May-Aug.)	F1	0.67	$0 \sim 1$
		Hamming Loss	0.32	$1 \sim 0$
		RMSD	0.04	>0
	West Pokot 2019 (Sep.-Dec.)	F1	0.43	$0 \sim 1$
		Hamming Loss	0.56	$1 \sim 0$
		RMSD	0.03	>0

C.2 Assessing model fit in Turkana

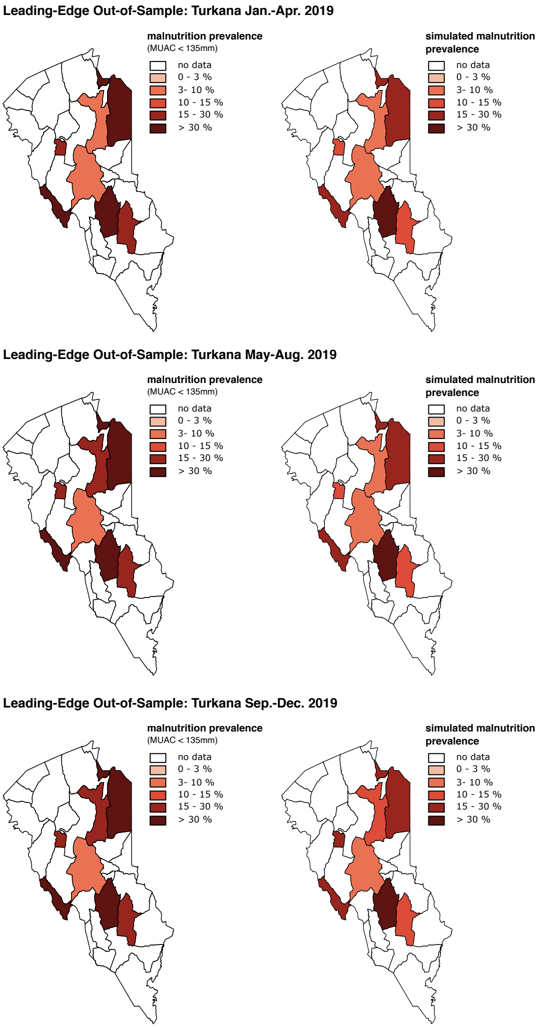

Figure S5: Turkana: 4-Month Leading-Edge Predictions (2019)

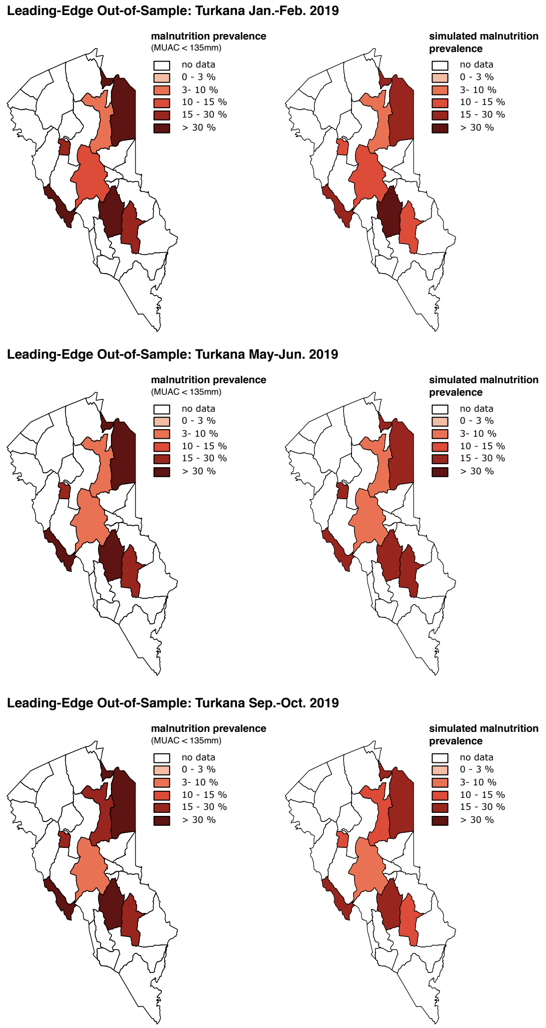

Figure S6: Turkana: 2-Month Leading-Edge Predictions (2019)

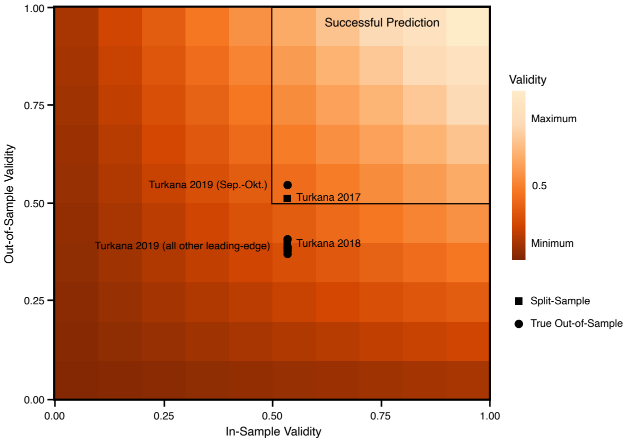

Figure S7: Turkana: Joint Validity Scale

Table S3: Turkana: Summary of Quantitative Model Performance Statistics

Sample	Region	Test Metrics	Results	Range
In-Sample	Turkana 2017-2018	F1	0.52	0 ∼ 1
		Hamming Loss	0.48	1 ∼ 0
		RMSD	0.14	>0
Out-of–Sample (Temporal Split)	Turkana 2017	F1	0.51	0 ∼ 1
		Hamming Loss	0.49	1 ∼ 0
		RMSD	0.10	>0
	Turkana 2018	F1	0.40	0 ∼ 1
		Hamming loss	0.60	1 ∼ 0
		RMSD	0.15	>0
Leading-Edge (2-months)	Turkana 2019 (Jan.-Feb.)	F1	0.41	0 ∼ 1
		Hamming Loss	0.59	1 ∼ 0
		RMSD	0.17	>0
	Turkana 2019 (May-Jun.)	F1	0.37	0 ∼ 1
		Hamming Loss	0.63	1 ∼ 0
		RMSD	0.15	>0
	Turkana 2019 (Sep.-Oct.)	F1	0.54	0 ∼ 1
		Hamming Loss	0.46	1 ∼ 0
		RMSD	0.07	>0
Leading-Edge (4-months)	Turkana 2019 (Jan.-Apr.)	F1	0.39	0 ∼ 1
		Hamming Loss	0.61	1 ∼ 0
		RMSD	0.16	>0
	Turkana 2019 (May-Aug.)	F1	0.40	0 ∼ 1
		Hamming Loss	0.60	1 ∼ 0
		RMSD	0.10	>0
	Turkana 2019 (Sep.-Dec.)	F1	0.41	0 ∼ 1
		Hamming Loss	0.59	1 ∼ 0
		RMSD	0.07	>0

C.3 Counterfactual experiments

C.3.1 Assumptions

Table S4: West Pokot: Assumptions for Counterfactual Experiment

Scenario	Model	Baseline Value	Assumption
Long Rains	stressors (X)	ability to store food based on 'average' year	reduce household ability by 20%
Economic	stressors (X)	not stressed	reduce household income by 50%
Household behavior	learning (λ)	optimized λ	household ability to learn is either constrained (low λ), enabled (high λ)

C.3.2 Projections

In order to arrive at the 4-months leading-edge predictions for the year 2020, we estimate the model based on data for the year prior and then project risk of acute malnutrition prevalence rates for April through July 2020. The corresponding Figure 7 in the main manuscript shows the empirical prevalence rates for 2019 up until March 2020 and three separate projections for the three scenarios described above (Table S4), where we provide the low and high λ as well as the baseline estimate for each. In Figure S8 we show the same projections but including two other wards covered by the NDMA data.

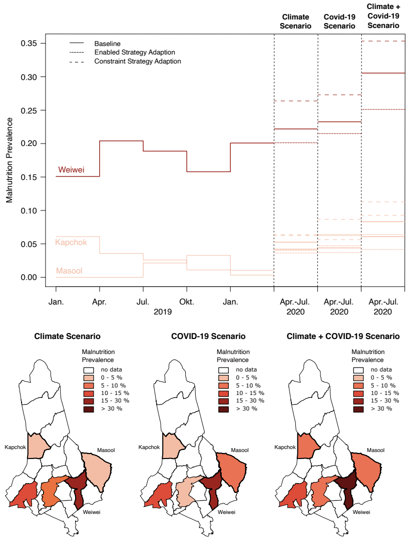

Figure S8: West Pokot: Counterfactual Scenarios for Alternative Wards (2020).

References

Bhavnani, R., Donnay, K., Reul, M. (2020). Evidence-driven computational modeling. *The SAGE Handbook of Research Methods in Political Science and International Relations*, 60.

Brown, M.E., Backer, D., Billing, T., White, P., Grace, K., Doocy, S., Huth, P. (2020). Empirical studies of factors associated with child malnutrition: highlighting the evidence about climate and conflict shocks. *Food Security*, 1–12.

Didan, K., Munoz, A.B., Solano, R., Huete, A. (2015). Modis vegetation index user's guide (mod13 series). *University of Arizona: Vegetation Index and Phenology Lab.*
